# A Transdisciplinary Approach Supporting the Implementation of a Big Data Project in Livestock Production: An Example From the Swiss Pig Production Industry

**DOI:** 10.3389/fvets.2019.00215

**Published:** 2019-07-04

**Authors:** Céline Faverjon, Abraham Bernstein, Rolf Grütter, Christina Nathues, Heiko Nathues, Cristina Sarasua, Martin Sterchi, Maria-Elena Vargas, John Berezowski

**Affiliations:** ^1^Vetsuisse Faculty, Veterinary Public Health Institute, University of Bern, Bern, Switzerland; ^2^Department of Informatics, University of Zurich, Zurich, Switzerland; ^3^Swiss Federal Research Institute, Birmensdorf, Switzerland; ^4^Federal Veterinary Office, Bern, Switzerland; ^5^Vetsuisse Faculty, Clinic for Swine, University of Bern, Bern, Switzerland; ^6^School of Business, University of Applied Sciences and Arts Northwestern Switzerland, Olten, Switzerland

**Keywords:** transdisciplinary, big data, pig, network analysis, food animal production

## Abstract

Big Data approaches offer potential benefits for improving animal health, but they have not been broadly implemented in livestock production systems. Privacy issues, the large number of stakeholders, and the competitive environment all make data sharing, and integration a challenge in livestock production systems. The Swiss pig production industry illustrates these and other Big Data issues. It is a highly decentralized and fragmented complex network made up of a large number of small independent actors collecting a large amount of heterogeneous data. Transdisciplinary approaches hold promise for overcoming some of the barriers to implementing Big Data approaches in livestock production systems. The purpose of our paper is to describe the use of a transdisciplinary approach in a Big Data research project in the Swiss pig industry. We provide a brief overview of the research project named “Pig Data,” describing the structure of the project, the tools developed for collaboration and knowledge transfer, the data received, and some of the challenges. Our experience provides insight and direction for researchers looking to use similar approaches in livestock production system research.

## Introduction

Big Data refers to data of varying formats and quality, produced at high velocity, and in large volumes, such that it cannot be easily processed with commonly used software tools (1). The potential benefits of Big Data approaches are well-documented in many industries. Use of Big Data in veterinary medicine was recently reported to be a promising way to improve animal health (2–8). However, these methods have not been broadly implemented in livestock production or health. Reports are limited to Big Data approaches being used for disease surveillance in companion animals (9, 10), within-farm analyses (7), and molecular epidemiology (4, 5). One reasons for this may be the relatively late adoption of computer and information technologies in the management of livestock production and health. This changing as the amount of data available in livestock production is growing rapidly due to increased availability of low cost data storage, adoption of precision livestock farming and development of new user-friendly computer and IT technologies. Large quantities of data are being generated, collected, and stored at all stages in animal production systems. Examples include animal movements, health, production and reproduction data, feed and water consumption, slaughter prices, and many others. Big Data approaches could be applied to much of this data to improve animal health, welfare, production efficiency and sustainability of both individual actors in the production system and the production system as a whole. Many animal health, disease, and management problems are not fully understood because it has not been possible to understand the whole picture of animal production. Big Data offers methods for developing a more complete picture and a better understanding of integrated animal production systems.

Implementing Big Data approaches in livestock production systems has significant challenges. A characteristic of these data is that they are created to manage livestock production, not to answer specific research questions. The “classical” scientific hypothesis-driven data creation and analytical skillset are not applicable to these data, changing the way scientific knowledge will be generated from these data. Since researchers and analysts are not directly involved in data creation they will be unfamiliar with the data. Understanding the data will require close collaboration among specialists from different disciplines and the providers or creators of the data (3). Creating knowledge will require the development of new analytical skills and methods, and the creation of an interdisciplinary environment which is in itself a challenging endeavor (11).

There are additional unique challenges associated with modern livestock production systems. Data about livestock populations is often collected by multiple independent private and public actors. Each actor has different interests, uses different data collection tools, and is involved in different strata of the production system. This heterogeneous mixture of actors creates challenges for data accessibility and integration. Data accessibility has been reported to be a major issue in public health (12). It is an even greater challenge in the private sector. In addition to privacy issues that have been intensified by the EU General Data Protection Regulation GDPR^1^, the private sector is subject to market pressures, as individual actors operate in a competitive environment. Communicating sensitive data may inadvertently provide an advantage to a competing organization, or the data provider may be at risk if the shared data are misused (13, 14). Sharing information among members of a production chain and researchers is not straightforward. Barriers to data sharing include lack of trust between partners, lack of transparency about the way the data are used, and lack of knowledge about the risks or benefits of data sharing (12–14).

Data integration, a well-known challenge in the field of data management, has become an even greater challenge in the era of Big Data (15). These challenges are also present in livestock production systems. Livestock data are difficult to integrate because the data are generated by numerous actors with different IT capacities using their own “in house” developed naming conventions (data semantics) and formats (data syntax). For example, one stakeholder may be a large trading company, which can allocate significant resources for data collection, storage and management. Other actors may be individuals, such as a veterinarians, for whom data collection may be a minor, side activity to their businesses. Close collaboration with data providers is essential for interpretation of individual data and for integrating multiple diverse data into a single, aligned data source.

Transdisciplinary approaches engage partners from multiple disciplines to create a holistic approach for solving complex societal problems. The goal of transdisciplinary research is to create new knowledge which contributes to societal progress by incorporating both scientific knowledge and societal perspectives (16–19). These approaches bring researchers from different fields together with society stakeholders to develop solutions valued by the stakeholders. Transdisciplinary approaches have potential for overcoming some of barriers to implementing Big Data approaches (20). Engaging stakeholders in the research process should help build trust between industry partners, ensure transparency, and provide benefits for data providers. All of these benefits should encourage data sharing among stakeholders, and between stakeholders and researchers. In addition, transdisciplinary approaches should facilitate data integration by supporting communication between data analysts and data providers.

Pig production industries in many developed countries are made of large integrated single owner systems. Switzerland's pig production system differs greatly from this model. The Swiss system has self-emerged as a decentralized, fragmented hierarchical network with cyclical components, and many small independent farms (21). Most farms own pigs only for a specific segment of the production chain. The Swiss pig industry is unique in its high animal welfare and health standards compared to other countries. Swiss law requires cage free farrowing, castration with anesthesia, and loose housing for pregnant sows. Health standards are high and the country is free of Classical Swine Fever, Aujeszky's Disease, Foot-and-Mouth disease, and Porcine Reproductive and Respiratory Syndrome (PRRS). Currently, actors in the Swiss pig production system use their data minimally, mostly for managing their own operations. These data are an untapped resource that could benefit the pig industry by contributing to a better understanding of complex health and production issues such as factors influencing meat and carcass quality, endemic health problems, or antimicrobial usage.

Transdisciplinarity and Big Data have both been reported to be increasingly important in research. However, practical recommendations and examples are largely unreported, especially for livestock production (16). This manuscript aims to help fill this gap by reporting the use of a transdisciplinary approach in a Big Data research project in the Swiss pig production industry. Our intent is not to comprehensively describe the project materials and methods and results, but rather to systematically document the process and lessons learned. We aim to provide insight and direction for researchers looking to use similar approaches in livestock production systems. We provide a brief overview of the research project, describe the structure of the project, the tools developed for knowledge transfer, the data received, and some of the challenges. We will discuss benefits of a transdisciplinary approach and provide some of the lessons we have learned.

## Project Objective

“Pig Data” is a 3 year transdisciplinary research project funded by the Swiss National Science Foundation (SNSF) that began in June 2017. Using Big Data approaches, this project explores the complex network of the Swiss pig industry and provides sustainable solutions for disease surveillance and real-time decision making. The project focuses on evaluating existing data sources, developing methods to collate them, and most importantly developing novel analytical and predictive methods that will produce information for decision making by the many diverse actors making up the production network.

## Project Partners

**Figure 1:**
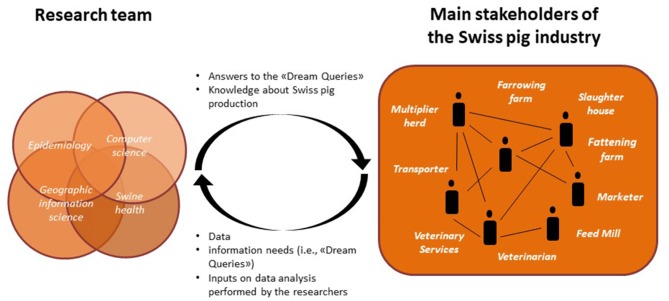
Pig Data project partners and data and information flows.

The “Pig Data” project brought together researchers from different research domains with key stakeholders in the Swiss pig industry (Figure 1). The research consortium is composed of four institutes working in different research domains: the Department of Informatics from the University of Zurich—UZH (computer science), the Swiss Federal Institute for Forest, Snow and Landscape Research—WSL (geographic information science), the Veterinary Public Health Institute of the University of Bern—UniBE (epidemiology), and the Swine clinic of the UniBE (porcine health management). The research task force is composed of two postdoctoral researchers and two PhD students. The task force is supported by five senior researchers, one from each of the research institutes involved in the project and one former employee of the UniBE. Key stakeholders and data providers in the Swiss pig industry were identified and invited to participate in the project before submission of the grant application. Key stakeholders include veterinarians, marketers, transporters, slaughterhouses, feed mills, and Swiss Veterinary Services. Stakeholders were selected because of their representativeness and impact in the Swiss pig industry. Every partner from the industry covers a significant part of the industry or market in his/her domain. The names and exact number of industry project partners cannot be reported for confidentiality reasons. All the stakeholders joined the project on a voluntary basis without receiving a monetary incentive.

## Transdisciplinary Collaboration

Specific tools were developed and implemented to promote collaboration and mutual understanding among the project partners. Needs for collaboration and mutual understanding varied depending on the project partners involved. To deal with the diversity, we defined and implemented rules and tools for collaboration among researchers and for collaboration between researchers and the stakeholders in the project.

### Collaboration Among Researchers

#### Start of the Collaboration

Two important initial steps in inter-disciplinary research are building a common vision of the problem and establishing a common vocabulary (22). To achieve these, we organized meetings at the beginning of the project to define collaboration rules within the research consortium. Members of each research institute presented their own research and collaboration practices, authorship, and venues for publishing and communicating scientific results. During these discussions, collaboration rules were defined in terms of meeting frequencies, communication tools, timelines, decision-making, authorship, and responsibilities. These rules guided research activities with the expectation that they could be modified when needs changed or upon request, and with agreement of the research consortium. To promote mutual understanding across disciplines and to minimize cultural barriers, personal goals, and expectations for project outcomes were also collected and shared among the researchers.

#### Collaboration Throughout the Project

The research consortium met face-to-face every 6 months for half a day to discuss the main project outcomes, next steps, and if necessary to re-evaluate each partners' goals and expectations. Bi-weekly meetings of the research consortium were organized to ensure regular and direct exchange of information on the project progress. These meetings were face-to-face for the research task force and mostly online for the other researchers. The research task force was responsible for the day to day work on the project and needed additional tools to be able to work together. Agile project management^2^ (23) is an adaptive project management approach derived from Software development. It consists of a succession of sprints, or iterations in which a specific piece of work is completed by the team in a defined time period. We adapted a simple Agile approach to the project. Sprints were organized to solve data management, data integration, and data analysis problems. In a typical sprint, a specific objective was defined before the sprint occurred, then the research task force met for a full day and worked together in the same room to achieve the defined objective.

#### Technical Tools Used to Support the Collaborative Work

Technical tools were selected and used to promote information and data exchange between researchers. A secure online storage space was created to share project-related documents and data files. To facilitate real-time communication, we used Mattermost^3^, an open-source, cloud-based, real-time communication software. Mattermost has several team collaboration features including persistent direct messaging channels that can be organized by self-defined topics. We also used Jupyter^4^, a browser-based interactive computational environment that allows collaborative implementation and execution of data science notebooks in programming languages such as Python and R. Jupyter allowed each research team member to have access to the centralized data store, explore, and analyse the data using various data analysis and data visualization libraries in both Python and R, without violating project data protection rules. These tools helped the task force to solve technical issues collectively and to effectively discuss data findings and methodologies used in the data analysis both during and after the sprints. Jupyter notebooks were used to document and share data analyses because code written in Jupyter notebooks allows annotation with text, images, and links to Web sites. Jupyter notebooks were essential for knowledge curation. In particular, the team was able to identify specificities and biases in the data that were subsequently corrected. Corrective actions were also reported in Jupyter notebooks in order to monitor implemented steps and to increase the reproducibility of the complete process.

### Collaboration Between Researchers and Stakeholders

#### Dream Queries

Stakeholders from the Swiss pig industry participated in the project during the grant writing process. They were asked to define research questions of primary interest to them with the understanding that the research task force would try to answer them when the project was operational. In return, they were asked to provide data to answer these questions and actively contribute to the overall research project. The questions defined by the stakeholders were called “dream queries” (DQs). Guidelines were developed to ensure the DQs would provide maximum benefit to stakeholders without putting them at risk for negative consequences due to data or information sharing. We selected DQs that required the analysis of multiple data sets to answer in order to demonstrate the value of combining different stakeholders data. Each DQ required approval by all stakeholders before being added to the list of DQs to be answered. If for any reason one stakeholder was uncomfortable with one DQ, they could anonymously reject the DQ with no justification and the DQ would be removed from the list of DQs to be answered. Project partners and stakeholders agreed to share the results of the analyses for each approved DQ with all other project members. Communication of individual DQ results beyond the project team was permitted only after approval by the complete project team, including all industry stakeholders. Fifteen DQs were defined and approved at the beginning of the project. Stakeholders were encouraged to submit additional DQs throughout the project. At the time of writing, 18 DQs have been approved (Table 1).

**Table 1:** List of DQs defined by the stakeholders of the Pig Data project at the time of writing.

**1**	What is the general relationship between genetic, breed, weather, transport condition (duration, loading time, etc.), and carcass quality (Lean meat, condemnation rate, slaughter weight, weight pork chop calculated, ham weight calculated, and ham weight calculated without bones, fat, and tendons)?
**2**	What is the general relationship between feed (specific type of feed, feed composition, or single ingredient), feed used in the nursery, feeding regimes, age at slaughter, antimicrobial usage, fattening performance, and carcass quality (Lean meat, condemnation rate, slaughter weight, weight pork chop calculated, ham weight calculated, and ham weight calculated without bones, fat, and tendons)?
**3**	Is there a significant difference in carcass quality between batches and/or between farms?
**4**	Is there a difference, especially in terms of carcass quality, between fatteners with assigned breeders (i.e., always same customers) or fatteners that can choose (and change) the breeders?
**5**	How pronounced is the difference in occurrence of condemnation of carcasses between homogenous (i.e., all pigs from the same breeding herd) and mixed batches?
**6**	Does the frequency of parturitions depend on the phase of the moon?
**7**	How does the temperature profile measured during a given summer impact the magnitude of “summer infertility syndrome” in sows and lower performance in fattening pigs during late summer and early autumn? And is there also an effect at later stages, e.g., health or fattening performance, in pigs born during “summer infertility”?
**8**	Is there a difference in fattening performance and revenue between batches of fattening pigs originating from the same breeding herd and mixed batches, i.e., with pigs from different herds?
**9**	How does the weight at which a pig enters the fattening phase (±1 kg) influence its later fattening performance, in other words what would be the optimal weight of a pig at housing-in?
**10**	What are causes or risk factors for the current unexplained accumulation of cases of hemorrhagic intestinal syndrome and arthritis in certain farms?
**11**	What is the influence of management, environment, and previous treatments on the occurrence of Mycoplasma hyorhinis as a herd-level problem?
**12**	Is there a seasonal pattern or do weather conditions influence the occurrence of hemorrhagic intestinal syndrome and cannibalism?
**13**	Does the quality scheme in which a farm is enrolled influence: (1) herd performance, (2) frequency of (a) gastro-intestinal disease in breeding/fattening herds, e.g., via scheme-dependent requirements like straw bedding; (b) lameness in fattening e.g., due to obligatory access to outdoor areas; (c) cannibalism due to particular climatic conditions in barns required in the scheme?
**14**	Do the labels have an influence on joint injuries/inflammations? What are the other factors having an influence on joint injuries/inflammations?
**15**	Is there an association between a specific type of feed, feed composition or single ingredient (minerals, additives etc.), and health parameters?
**16**	How is the Swiss swine industry organized? In particular, what is the proportion of fatteners with assigned breeders vs. those enrolled in a free market scheme?
**17**	What are the regional factors (geographical regions of Switzerland, but also agricultural zones—valley zone, mountain zone, etc.) influencing carcass quality?
**18**	Is there any differences in terms of slaughter age between fattening farms?

#### Collaboration Throughout the Project

Regular informal contact between the research consortium and industry stakeholders was maintained to improve data literacy, discuss outputs of analyses, ensure mutual understanding of data and to transfer knowledge. The importance of stakeholder opinions and suggestions was regularly emphasized and stakeholders were frequently asked to provide feedback on project activities. Specific communication tools and information products were developed in collaboration with stakeholders. Progress of the project was communicated in bi-annual newsletters and face-to-face meetings. These alternated so that every 3 months, stakeholders received a newsletter or attended a project meeting. The newsletter was two to three pages long and reported general information about the project (e.g., scientific conferences attended, key results obtained). During project meetings, the most recent results were presented and discussed among project partners. Informal gatherings were organized after these meetings to allow participants the opportunity for further discussion and for relationship building. Main results of the DQ analyses were presented in the newsletters or during the face-to-face meetings. Because the DQs and their answers were often complex, the research consortium also distributed short scientific reports to stakeholders. These reports contained detailed information on data selection and cleaning, assumptions, the full results and a summarized interpretation of the results. A draft of each report was sent to all stakeholders for comments and suggestions. Meetings were organized upon request from individual stakeholders to discuss the results in more detail.

## (Big) Data

### Data Confidentiality and Data Sharing Agreements

Data confidentiality was a primary concern for all project partners. Data sharing agreements were signed between the University of Bern and each stakeholder. Each data sharing agreement contained common rules and additional rules to meet individual stakeholder needs. The common rules ensured that stakeholders retained ownership and control of their own data and that only the research consortium had access to the data. To ensure stakeholders had control of information produced from their data, all stakeholders were required to approve information before it was communicated outside the project. To limit the risk of violating confidentiality, stakeholders were asked to remove confidential information from their data before sending it to the project. However, some of the data still contained sensitive information. To further reduce the risk of privacy breaches, data were stored in encrypted libraries.

### Data Collection

At the start of the project each stakeholder was interviewed to collect information about their data and to develop a working relationship between stakeholders and project researchers. To develop a process for transferring data, stakeholders were asked to describe the characteristics of their data (nature, format, variable values), the processes for collecting their data, and if technically and easily possible to provide a small sample of their data. Each stakeholder provided names of contacts for answering technical questions and interpreting data. Since veterinary stakeholders used proprietary practice management software for data collection and storage, meetings with the two companies providing their software were arranged. Extracting data from veterinary practice management software is an unusual business activity and both software providers were required to develop additional software to extract their data.

Initial data extractions were completed by all stakeholders within the first 6 months of the project. This data was historical, covering the period from 2011 to mid-2017. The data collected were very diverse and included pig transport, meat quality, reproduction, feed, health, and climate data (Table 2). Stakeholders were asked to provide updated data every 1–2 months by exporting it to a dedicated folder on a locally run Dropbox clone (Seafile)^5^. All data were created for stakeholder business purposes (e.g., organization of animal transports, issuing of invoices). The research consortium had no control over any of the data collection processes.

**Table 2:** Data collected within the Pig Data project.

**Data type**	**Data provider**	**Dataset description**	**Example of data available**
Laboratory data	Swiss veterinary services	Data contain information from 25 accredited laboratories involved in the diagnosis of the 70 notifiable epizootics in Switzerland	Sampling date, farm ID, zip codes, report date, who did the sample, which laboratory did the test, method, result, etc.
Health and treatment data	Veterinarians	Data contain information on pig farms visited by swine veterinarians	Visit date, treatment delivered, symptoms observed, etc.
Reproduction data	Marketers	Data contain information on reproduction performance	Number of gilts/sow, insemination, farrowing rate, insemination date, abortion, piglets loss, etc.
Fattening performances	Marketers	Data contain information on fattening performance	Number of fattening pigs, average daily gain, feeding system, etc.
Feed data	Feed mill	Data contain information on feed sold to pig farms	Date of feed delivery, type and amount of feed, receipt of the feed, etc.
Swiss animals movement database	Swiss veterinary services	Data contain official notifications of pig movements in Switzerland	Date of transport, date of notification, farm ID of departure, and arrival, number of pigs, etc.
Logistic transport data	Transporters	Data contain detailed information on pig transports	Date of transport, truck ID, sequence number, number of pigs, farm ID of departure and arrival, arrival time, etc.
Billing data	Marketers	Data contain information on pigs sold by the marketer	Date of transport, zip codes, number of pigs, farm ID of departure and arrival, weight of pigs, etc.
Meat quality data	Slaughterhouses	Data contain detailed information on pig meat parameters assessed by slaughterhouses	Date of slaughter, animal ID, warm weight, meat mass, fat score, carcass classification, etc.
Full carcass condemnation	Swiss veterinary services	Data contain information on slaughtered pigs and full carcass condemnation from all over Switzerland	Date of control, date of carcass release, number of pigs, reason for condemnation, slaughterhouse ID, farm ID etc.
Partial carcass condemnation	Slaughterhouses	Data contain information on partial carcass condemnation such as	Date of slaughter, Number of pigs, reason for condemnation.
Climate data	MeteoSwiss	Data contain measurements from land-based stations of one or more ground-level monitoring networks.	Minimal temperature, maximal temperature, humidity, etc.

### Central Data Repository

Data sets were in multiple data formats (e.g., old databases like dBase, XML, or Excel files). To integrate these data in a central data repository, all datasets were automatically processed and transformed into a homogeneous interoperable format (CSV). We transformed each data set individually, aligning them syntactically, and semantically in a relational database. The data base contained tables for all data sets. Data were linked by a standardized holding identifier (a farm ID number), which reduced the number of schema and data heterogeneities (e.g., homogenizing date formats). Researcher access to the database was provided by an SQL query endpoint. The data provenance was modeled in the database. We maintained a versioned repository of data transformation and injection scripts, allowing us to reproduce our steps at any time.

### Data Validation

Each dataset was thoroughly described and assessed in order to validate the data, understand their meaning, and identify important data gaps and quality issues. For some data variables, consistency was assessed by comparing information available in different datasets. For example, we were able to compare the arrival time at a slaughterhouse that was reported by pig transporters to the arrival time reported by the slaughterhouse. We also compared the number of pigs transported in a single shipment that was reported in each database. Data varied in quality between data providers and between data variables within individual datasets. Some data variables had to be excluded from analyses because of the large number of invalid entries (e.g., single pigs with a body weight >1,000 kg), or numerous missing values. Other types of inconsistencies between data providers were identified. For example, the time of slaughter reported by the slaughterhouse was in some cases earlier than the time of arrival of pigs at the slaughterhouse as reported by the pig transporter. In some cases there were differences in the number of pigs in a single transport when it was reported by the transporter, the national animal movement database or the slaughterhouse. Understanding the data and identifying the main data quality issues required deep data exploration and frequent communication with data providers.

The biggest challenge for data analyses was the absence of individual pig identifiers in many datasets. A lot of data was available at the farm or batch level, but there was insufficient data to allow tracking of an individual pig during its entire life (i.e., from the farrowing farm to the slaughterhouse). The second most important challenge related to the characteristics of the data from veterinary practitioners. These data were recorded in free text and were very heterogeneous (e.g., words written in High German and Swiss German, with numerous abbreviations and typing errors). Text mining tools were used to structure these data for subsequent analysis. Records relating to pigs were separated from those for other species using 27 words related to swine (e.g., “schwein,” “muttersau,” “ferkel,” “porci,” etc.) and their different orthographical and plural forms. Veterinary records from 15,562 unique visits related to 243 pig farms were extracted for further analysis. Each farm visit had one anamnesis (history), but often had multiple entries for treatments, medical activities, or had multiple comments made by the attending veterinarian. Text mining tools were also used to automatically identify text related to drug delivery, medical activities (e.g., on-farm visit, castration, surgery), product delivery (e.g., antiseptic, gloves), or clinical observations made by the veterinarians. Sixty percent of the entries were identified as having a drug delivery and drugs recorded were matched with the anatomical therapeutic chemical classification system for veterinary drug products in Switzerland (ATCvet)^6^. The percentage of mismatches (i.e., a drug recorded in the data not associated with its correct ATCvet code) was low (0.07% for antimicrobial drugs). These results are encouraging, but at the time of writing, identifying diseased animals remains a challenge that has not yet been overcome.

## Data Analysis

### Understanding the Complex Swiss Pig Production Network

One of the initial goals of the Pig Data project was to understand and model the structure of the Swiss pig industry. Our first step was to fully describe the pig transport network using the Swiss official animal movement database. We then analyzed the topology of the Swiss pig transport network using tools from social network analysis and assessed the implications of various network characteristics on the spread of infectious diseases. Since we had additional pig movement data from pig transporters we compared the information from the two data sources. We assessed the effect of additional information about the sequence of pig transports (from pig transporters) on the topology of the transport network. The work was based on the official animal movement database in Switzerland and a sample of transport data from one of our industry partners. To our knowledge this is the first published report in which a single livestock transport network was evaluated using two different data sources and it highlights the value of combining data from different sources. For more details regarding, please see Sterchi et al. (24).

### Answering Dream Queries

At the time of writing, six out of 18 DQs have been answered (i.e., DQs 1, 3, 4, 5, 8, and 16). Dream Queries 1, 3, 4, and 5 were answered using a combination of weather data, data from multiple stakeholders and the output of the analyses for DQ 16. We estimated the effect of numerous animal transport factors on meat quality using data from more than 500,000 pigs, 5,000 transports, and 600 farms. Dream Query 16 was answered by combining the output of network modeling (see Understanding the Complex Swiss Pig Production Network above) with data from one of our industry partners, on a subset of Swiss fattening farms. Results of these DQ analyses were presented to our industry stakeholders. All stakeholders responded positively and expressed their satisfaction with the results in terms of the value of the information created. Some of the stakeholders even provided additional data to improve DQ answers. The results of these DQs are planned to be published separately in scientific and/or professional journals. Time constraints and data quality issues will prohibit answering all DQs. It will not be possible to answer dream queries 9 and 18 because of the absence of individual pig identifiers in the data. We will not be able to answer dream queries 10–15 because of the unstructured format of the veterinary practice data. The amount of time and work required to answer each dream query is considerable, as most of the dream queries are independent and detailed epidemiological studies on their own. The project has limited resources and this will affect our ability to answer additional dream queries before the end of the project.

## Discussion

### Collaboration Among Researchers

#### What Worked

Having an inter-disciplinary research team was essential for much of the success achieved in the Pig Data project. For example, aligning different datasets using pig farm characteristics required knowledge about the structure of the Swiss pig industry, potential risk factors of interest, geographical information systems, and computer science. The adoption of an Agile project management approach with supporting software tools facilitated transdisciplinary collaboration. Sprints and Jupyter notebooks provided the greatest benefit. The Pig Data project is a complex and dynamic project. Advance planning of tasks was often not possible as new problems and questions frequently emerged. Sprints were easily adapted to the project because they allowed flexible planning and fast progress. The use of Jupyter notebooks allowed researchers working on the data to record the rationale for their data cleaning, manipulation and analyses in the same place as the functional code. This supported collaboration by allowing other researchers using the same datasets for other analyses to quickly and easily understand previously written code. Improvements to the code could be explained, justified, and easily communicated to others working on the data. The collaborative benefits of Agile project management have been reported (25, 26). Our experience demonstrates that agile project management can be beneficial in transdisciplinary research projects where high flexibility and close collaboration between researchers is required.

#### What Didn't Work

The transdisciplinary collaboration in the project could have been improved. For example, we did not fully implement an Agile project management approach. We only adapted some parts (for example sprints) to our needs. We believe that adopting a more complete Agile management approach could have increased the efficiency of the research task force. We encourage other researchers managing complex research projects to consider more complete adoption of Agile management methods. Knowledge management, curation and transfer in a rapidly evolving environment such as the Pig Data project was challenging, and could have been improved. Wiki-based approaches may have been beneficial, but they require an active community that regularly contributes to knowledge curation. This would have been a challenge in our project because most of the project team members were working on a limited time budget. As in other transdisciplinary projects (22, 27), the Pig Data research consortium did not completely overcome the challenges of communicating across disciplines. Building a common vocabulary with a shared understanding of a problem and a joint set of objectives should be done at the beginning of a project and updated frequently during the project (22). We cannot sufficiently stress the importance of regular face-to-face meetings to improve and maintain effective communication. Allocating sufficient time for communication activities is essential. When we designed the project, we did not allocate any time specifically for this purpose and we had to attempt to do this in parallel with other large mandatory tasks. This crucial part of transdisciplinary work is rarely valued in scientific publications (28), making it difficult to justify allocating time and resources for these activities to funding agencies and project partners. However, dedicating generous amounts of time for team building in transdisciplinary projects is essential and we encourage researchers to request funding for this purpose and granting agencies to provide the needed funding.

### Collaboration Between Researchers and Stakeholders

#### What Worked

Among the greatest strengths of the Pig Data project were the continuous communication and collaborative relationships between project researchers and Swiss pig industry stakeholders. Stakeholder engagement was essential for properly aligning and interpreting data and producing meaningful results. We recognized the importance of stakeholder relationships and planned activities to build and strengthen them. Stakeholders became strongly committed early in the course of the project. They attended meetings, quickly responded to emails, regularly updated data, spontaneously provided other contributions to the project (i.e., new DQs, datasets, ideas), and expressed interest in being involved in the data analysis. The DQs played a key role in this success as they allowed stakeholders to clearly see how they could benefit from the research project. Regular face-to-face meetings, email communications, and newsletters contributed to building trusting and transparent relationships between researchers and stakeholders. The informal gatherings following official face-to-face meetings were very important for building personal relationships between project partners. They provided an opportunity for open and frank dialogue, and allowed us to collect questions or remarks that stakeholders may not have been comfortable sharing during meetings. Frequent solicitations by the research team to provide feedback on project activities may have contributed to empowering the stakeholders to more fully participate in the project, and to maintain their engagement throughout the project. Empowering stakeholders to identify and solve their own problems is a core concept of participatory epidemiology, a growing field in animal health (29). Participatory epidemiology is often considered applicable only in developing countries (29). We believe that the Pig Data project is a good illustration of the feasibility and benefits of participatory approaches in developed countries.

#### What Didn't Work

Collaboration between researchers and stakeholders was good overall, but there were challenges that could have been better dealt with. Language was a challenge. Communication between stakeholders and researchers was split between English and German. We believe this was a moderate problem for industry stakeholders. Switzerland has four official languages, and industry stakeholders are accustomed to switching between languages. It was more of a problem for some of the international researchers on the team who did not have a high level of competency in German. Communication is always challenging in collaborative projects and whenever possible, using a single common language would be preferable. The project would have benefited with greater stakeholder involvement in data analysis, data linkage and knowledge transfer. Greater input from stakeholders would have allowed us to better adapt communication products to stakeholder needs. For example, we were not able to provide stakeholders with personalized outputs. Without stakeholder input, communication was driven primarily by researcher interests which were focused on the data, analyses and results that could be published. One reason for failing to engage stakeholders in communication was the shortage of human resources in the project. When planning the project we did not fully comprehend the large amount of work required and did not include sufficient human resources in our grant application. The research team did not have the resources to develop a full transdisciplinary collaboration which included communication, and at the same time to deal with Big Data issues, answer complex DQs, and perform research work. We reduced the work load by recruiting additional students to work on some parts of the project and by reducing the frequency of data transfers from stakeholders. However, the large workload remained a challenge for the research team. Transdisciplinary work allows the development of many unique skills and provides opportunities to achieve research goals that would not be possible using more conventional research approaches. However, it can be overwhelming for young researchers who are required to meet the requirements for more traditional academic excellence and at the same time deal with the additional work required for a transdisciplinary research project. Career progression following this path is not easy for young scientists (28, 30). We recommend that all researchers planning transdisciplinary research comprehensively explore and understand the trade-offs between research and career development before writing a grant to fund their research.

### Big Data Approaches in the Swiss Pig Industry

#### What Worked

Basing the research consortium and database in a university was essential. Academic institutes are neutral third parties with no commercial or regulatory interest in the data. Working in academia allowed us to build a safe space with a clear boundary between the project and both national disease control authorities and competing businesses. This allowed the research team to be completely transparent within the boundaries of project and provided security necessary to reduce data and information sharing risks for the data providers (31). A transdisciplinary approach was essential from the onset of the project. We were not able to provide stakeholders with monetary compensation for participation in the project. It would have been very difficult to convince them to share their data with us without engaging them in the project and demonstrating that we could provide valuable information that was not attainable from their individual datasets. The data collected, the skillsets and methods provided by the research team, and the collaborative relationships that were developed provided new learning opportunities for researchers and stakeholders. For example, the relationships between piglet producers and fattening farms were expected to be complex, but their complexity greatly exceeded stakeholder expectations (24). Text mining tools enabled automatic classification of drug sale data into different sub-categories. With this data we were able to identify different prescription patterns between veterinary practices and between farms that were previously unknown (data not shown). We were able to answer a large number of DQs which provided useful previously unknown information to the stakeholders. The partial overlap between some data sources combined with the inputs of the data providers enabled us to assess the quality of each data source and provide some insight into the uncertainties of the research outputs. The Pig Data project itself is a significant research achievement. To our knowledge it is the first project reported that combines large amounts of diverse data from public and private livestock industry stakeholders in one single data store for one purpose.

#### What Didn't Work

An important issue early in the project was the absence of data descriptions and information about the quality of most datasets. This made data integration and interpretation very difficult and time consuming. Since the research team did not know how stakeholders created and managed their data, it was necessary to contact individual stakeholders every time an anomaly was found in a dataset. The free text format of veterinary practice data made it difficult to identify specific diseases or syndromes of interest. Text mining tools have been reported for extracting clinical data from electronic patient records created using veterinary practice management software. However, these reports are only for companions animals (9, 32, 33). Companion animal data are consistently recorded at the individual animal level. This was not the case for our pig veterinary data which were mostly recorded at the herd level restricting our analyses to assessment of herd level patterns. Companion animal electronic veterinary records contain both animal health information and information for client invoicing. The information in our pig veterinary practice data mostly related to client invoicing, making it difficult to extract useful data about pig diseases, symptoms of disease, and other data about pig health. Data cleaning and pre-processing is widely recognized as one of the most time-consuming steps the Data Science process (34). We confirmed this in our project. One of most important lessons we learned is that it is very easy to underestimate the amount of time and effort needed for processing raw data and exploratory data analysis. It is difficult to accurately estimate the resources needed for these activities during the grant writing and planning stages of a project. We recommend that when planning projects that include a variety of diverse data providers, extra efforts should be made by project planners to understand the quality and variability of these data. Obtaining data samples and conducting interviews of data providers are essential to accurately plan and budget for the needed time and resources.

### Limitations

An important limitation not mentioned above, was not including farmers directly in the project. Farmers are excellent sources of practical agricultural knowledge and they could have contributed to the assessment of biases present in the data, and the interpretation of the results of analyses. They would have also provided more detailed information on farm characteristics and practices. We chose not to include farmers to simplify the project. Finding farmers that complied with inclusion and exclusion criteria for project partners would have been difficult or impossible. There are many small farmers using many different data collection approaches in the diverse Swiss pig production system. Including farmer data in the project would have made data integration much more complicated, difficult, and time consuming, adding even more work to our resource limited research task force. However, one of our project goals is to develop communication products for the entire Swiss pig production chain and we hope to involve farmers in subsequent projects. Involving the entire Swiss pig production community would have been essential for fully understanding the pig production system. Our recommendation for researchers planning similar large scale projects is to include representatives of all stakeholder groups.

### Generalization of the Approach

A transdisciplinary approach has been reported to help overcome many important barriers to data sharing in agriculture production chains, including lack of trust between stakeholders, lack of transparency about the way data are used, and lack of knowledge about the benefits and risks associated with data sharing (12–14). This approach has been reported to have potential for solving Big Data accessibility issues (6) and should be generalizable to other livestock production systems and other countries. Transdisciplinary approaches also have well-known challenges for project design and management (27) and these may also be generalizable to other projects and countries. Our project was designed as a traditional research project, not as a transdisciplinary project, and the structure of our project differed considerably from the ideal or typical conceptual model for a transdisciplinary research project (27). In our experience classical project management methods are not well-adapted to complex transdisciplinary research projects. We recommend that transdisciplinary project design methods should be carefully considered when planning and implementing large projects.

To date, the biggest success of the Pig Data project is demonstrating to Swiss pig industry stakeholders and the research team that the large volumes of highly varied data that are continuously produced in the Swiss pig production system have value. We demonstrated that new, interesting and useful information can be created when these data are integrated and analyzed by a collaborative multi-disciplinary analyses team working closely with industry stakeholders. We believe that this is generalizable to other livestock production systems in other countries. Many of the challenges will also be generalizable. Data from multiple stakeholders in large livestock production systems are not easy to deal with. The data are generally not created to combined or for research purposes. They are secondary data, created for managing individual businesses in the production network. We expect this problem will be common in other livestock production systems and in other countries. Involving data providers' directly in data cleaning, pre-processing, and interpreting of analysis results was essential for our project and will likely be essential in similar projects in other livestock production systems. Stakeholder engagement made many of time-consuming and difficult tasks much easier, and reduced error (34). We agree with (35), and argue that involving data providers in all parts of the project was critical for ensuring the reliability of the information produced. This project demonstrated that transdisciplinary approaches can help to address one of the major challenges of working with Big Data: establishing the value of the results obtained from large quantities of highly heterogeneous data (6).

### The Future

Sustainability of large complex collaborations such as the Pig Data project is not a trivial undertaking. Developing a strategy for transitioning the Pig Data project into a sustainable network that benefits the Swiss pig production community into the future is a goal of the Pig Data project. As we approach the end of the research grant period, it will be necessary to think creatively about solutions for continuing the collaboration. We do not expect this to be simple or easy, and there are (to our knowledge) no previous studies published that we turn to for guidance. Our approach will be participatory and transdisciplinary engaging all stakeholders in the discussion and planning. The Pig Data project was designed as an applied research project producing outputs that have practical application in the pig industry. We expect that a second project emerging from this one will be even more applied, and for this reason may require more input from the stakeholders and the addition of researchers with more applied research interests.

## Conclusion

The Pig Data project has demonstrated that Big Data approaches can be successfully implemented in livestock production systems using transdisciplinary methods. We have shown that valuable new information can be created from the highly variable data produced by livestock production system stakeholders. The information created has been shown to be valued and useful to both stakeholders and researchers. We have demonstrated the importance of adopting an inclusive, participatory approach that engages a wide range of researchers and industry stakeholders. Our experiences provide some insight and direction for researchers looking to use similar approaches and we hope that our work will promote the implementation of both Big Data and transdisciplinary approaches in livestock production system research. At the time of writing, the Pig Data project is not yet completed, but it can already be considered a success in terms of the quantity of data collected, the outcomes produced and the return on investment for industry stakeholders and researchers.

## Author Contributions

JB, CN, HN, AB, and RG were the co-PIs of the project. CF contributed to the project coordination and interaction with the stakeholders. CS, MS, M-EV, and CF collected, integrated, and analyzed the data. CF drafted the manuscript. JB, CN, HN, AB, RG, CS, MS, and M-EV critically reviewed the manuscript. All the authors approved the final version and contributed to the design of the study.

### Conflict of Interest Statement

The authors declare that the research was conducted in the absence of any commercial or financial relationships that could be construed as a potential conflict of interest.

## Footnotes

^1^General data protection regulation https://eugdpr.org

^2^Agile Manifesto http://agilemanifesto.org/

^3^Mattermost https://mattermost.com/

^4^Project Jupyter http://jupyter.org/

^5^SeaFile https://www.seafile.com/en/home

^6^ATCvet https://www.whocc.no/atcvet/
